# Functional Trajectories Following Clinically Selected Management of Concomitant Proximal Humerus Fractures in Geriatric Hip-Fracture Patients: A Retrospective Cohort Study

**DOI:** 10.3390/jcm15218361

**Published:** 2026-10-27

**Authors:** Borja Lara-Galdón, José María Hernández-Mateo, Lourdes Prats-Peinado, Álvaro Guzmán-Sánchez, Carmen Begara-Bretón, Mikel Aburto-Bernardo, Ángel Pérez-Martín, Fausto López-Mombiela

**Affiliations:** 1 Department of Orthopaedic Surgery and Traumatology, Hospital General Universitario Gregorio Marañón, 28007 Madrid, Spain; 2 Universidad Complutense de Madrid, 28040 Madrid, Spain; 3 Shoulder and Elbow Unit, Department of Orthopaedic Surgery and Traumatology, Hospital General Universitario Gregorio Marañón, 28007 Madrid, Spain

**Keywords:** hip fracture, proximal humerus fracture, Barthel Index, geriatric patient, functional recovery, treatment selection

## Abstract

**Background/Objectives:** Concomitant hip and proximal humerus fractures create a demanding rehabilitation scenario in older adults. We described unadjusted functional trajectories following clinically selected proximal humerus management and explored the relationship between baseline independence and treatment allocation. **Methods:** We retrospectively reviewed 4776 hip-fracture presentations (2011–2023); 55 patients aged > 65 years had synchronous proximal humerus fractures. All hip fractures were surgically treated; 13 proximal humerus fractures were managed operatively and 42 non-operatively. Barthel Index was assessed before fracture, at 30 days, and at six months. **Results:** Mean pre-fracture Barthel scores were 98.5 ± 3.8 and 79.5 ± 20.8 in the operative and non-operative groups, respectively. At 30 days, scores were 85.4 ± 6.9 versus 60.5 ± 23.9; at six months, scores were 93.5 ± 5.2 versus 70.2 ± 25.0. Mean absolute change from baseline at six months was −5.0 versus −9.3 points. **Conclusions:** Patients selected for surgery were more independent before injury and remained more independent at follow-up. Marked pre-injury differences and confounding by indication prevent any inference about the independent effect of proximal humerus surgery.

## 1. Introduction

Hip fracture in older adults is a sentinel event associated with loss of autonomy and substantial rehabilitation needs. Functional recovery is heterogeneous and is influenced by pre-fracture activities of daily living, cognition, comorbidity, nutritional status, and the ability to mobilize early [1,2,3,4,5]. The Barthel Index is widely used to quantify basic activities of daily living and has been specifically studied as a measure of functional recovery after osteoporotic hip fracture [1].

Recovery after hip fracture is therefore better understood as a return toward an individual functional baseline than as attainment of a single postoperative score. A patient who was previously independent and a patient who already required assistance may have different rehabilitation goals despite sustaining similar fractures. Pre-injury function also helps clinicians and families interpret the practical meaning of residual disability. Measuring the same activities before and after injury provides a useful description of this course, although it does not by itself separate the contributions of the hip, shoulder, and general health to recovery [1,2,3].

A synchronous upper-extremity fracture can further complicate recovery because transfers, walking aids, and progressive ambulation often require effective use of the arms. A systematic review and meta-analysis found worse rehabilitation-related outcomes in patients with combined hip and upper-extremity fractures, while recent large cohorts confirm that these combined injuries identify a clinically vulnerable subgroup [6,7,8,9]. Proximal upper-extremity injuries may be particularly relevant because they directly compromise the shoulder girdle and the ability to load the arm during mobilization [7].

The combination creates potentially competing rehabilitation requirements: the lower limb needs progressive mobilization, while the injured shoulder may require protection. Even when loading through the operated hip is permitted, pain or restrictions affecting the arm may limit how that permission can be translated into safe transfers and walking. Conversely, maintaining some shoulder movement does not necessarily mean that the arm can support body weight. These distinctions make assessment of the whole rehabilitation pathway particularly relevant when considering treatment of the proximal humerus [4,5,10].

Evidence directly addressing concomitant hip and proximal humerus fractures remains sparse. Ganta et al. compared operative and non-operative humeral treatment in this setting and reported that surgically treated patients were younger and more often community ambulators at baseline, with a non-significant trend toward return to baseline ambulation at 6 months [10]. This illustrates both the potential rehabilitation rationale for surgery and the major problem of treatment-selection bias.

Management of proximal humerus fractures in older adults is itself controversial. Contemporary reviews and guidelines emphasize individualized treatment [11,12,13]. Randomized evidence including NITEP and PROFHER does not support routine surgery for all displaced fractures [14,15,16], while recent trials and meta-analyses suggest that reverse shoulder arthroplasty (RSA) may improve selected functional outcomes in complex fractures, at the cost of procedure-specific complications and reinterventions [17,18,19,20,21,22,23]. Non-operative care and rehabilitation remain appropriate for many older patients [24,25,26], and frailty may materially influence prognosis [27].

Operative treatment also comprises distinct strategies rather than a uniform intervention. Internal fixation preserves the native humeral head, whereas hemiarthroplasty and reverse shoulder arthroplasty replace the joint with different functional and biomechanical expectations. Trials comparing these procedures address a different question from trials comparing surgery with non-operative care. Their findings must therefore be interpreted according to fracture pattern, the procedure studied, and the outcome measured; a shoulder-specific functional advantage cannot automatically be translated into improved walking or global independence [14,17,28,29].

In patients with two simultaneous fractures, baseline autonomy has a dual role. It is relevant to prognosis, but it may also influence whether the additional shoulder operation is considered worthwhile and feasible. Consequently, patients selected for surgery may start with a greater likelihood of functional recovery before any shoulder intervention occurs. Describing these selection patterns alongside subsequent recovery is clinically informative, provided that the resulting group differences are not interpreted as evidence of treatment efficacy. This distinction is especially important in small observational cohorts that cannot adequately capture or adjust for the full clinical decision process [10].

The Barthel Index provides a pragmatic measure of global independence. In the conventional 0–100 interpretation, scores of 91–99 represent slight dependence and 100 independence [30,31]. Accordingly, a value > 90 can describe preserved or near-independent basic activities of daily living, but it has not been validated as a threshold for proximal humerus surgery.

The objective of this study was to describe unadjusted functional trajectories following clinically selected operative or non-operative management of synchronous proximal humerus fractures in older patients undergoing hip-fracture surgery. We also describe the pre-fracture functional differences associated with treatment selection. This observational study was not designed to estimate the comparative effectiveness of proximal humerus surgery.

## 2. Materials and Methods

### 2.1. Study Design and Population

This retrospective single-center cohort included patients older than 65 years with synchronous hip and proximal humerus fractures treated between January 2011 and December 2023. Synchronous fractures were defined as injuries involving both regions from the same fall, diagnosed during the same hospital admission; injuries resulting from separate events were excluded. All hip fractures were surgically treated. Of 4776 screened hip-fracture presentations, 55 patients were included (13 operative and 42 non-operative humerus treatment). Barthel Index measurements at pre-fracture baseline, 30 days and six months were available for all 13 patients in the operative group and all 42 patients in the non-operative group at each assessment. Mortality was assessed to 12 months; two deaths occurred in the non-operative group at months eight and eleven, after the six-month functional endpoint.

### 2.2. Treatment Allocation

Patients were classified according to management of the proximal humerus fracture: operative (*n* = 13) or non-operative (*n* = 42). Allocation was clinical rather than randomized and incorporated fracture morphology (Neer/Hertel criteria), comorbidities, general condition, expected functional demands, and pre-injury functional status. Baseline function was therefore part of the process that created the groups, introducing confounding by indication and treatment-selection bias.

### 2.3. Functional Assessment and Rehabilitation

Pre-fracture functional status was assessed using the Barthel Index by the Geriatrics team during the index hospital admission as part of routine geriatric assessment. The assessment referred to independence before the traumatic event and was obtained by direct patient interview whenever feasible; if the patient could not provide reliable information, a close family member or primary caregiver was interviewed. For this study, the contemporaneously documented pre-fracture scores were extracted from the medical record rather than reconstructed by the investigators. Barthel Index was also assessed at 30 days and six months. Hip-fracture rehabilitation emphasized early mobilization, with postoperative weight-bearing initiated when clinically and radiographically appropriate. After operative treatment of the proximal humerus fracture, elbow movements were permitted immediately and shoulder pendulum exercises were introduced early, with progression according to the surgeon’s assessment. In the non-operative pathway, sling immobilization was generally used during the first week, with active hand, wrist and elbow movements; gentle pendulum and passive shoulder mobilization were typically introduced during weeks 1–2 according to pain and stability. The sling was progressively discontinued around weeks 2–3 as active-assisted shoulder exercises began. Active shoulder mobilization was progressed during weeks 4–6 following clinical and radiographic assessment, and strengthening and partial upper-limb loading were considered from weeks 6–8 when healing permitted. Functional loading was subsequently increased during weeks 8–12 according to consolidation and tolerance. During early hip-fracture rehabilitation, transfers and walking aids were adapted to avoid loading the injured upper limb. These time frames describe the routine treatment pathway rather than individually documented adherence for every patient.

### 2.4. Outcomes

The primary outcome was functional trajectory measured by the Barthel Index. Secondary outcomes included length of hospital stay, readmission, mortality, and the clinical variables available in the original dataset. The Barthel Index was interpreted as a global measure of autonomy rather than a shoulder-specific outcome.

### 2.5. Statistical Analysis

Continuous variables are reported as mean ± standard deviation, and categorical variables as *n* (%). Between-group Barthel scores at each time point were compared using two-sided Welch t-tests, allowing for unequal variances and group sizes. Individual absolute changes from pre-fracture baseline to 30 days and six months were summarized using mean differences and descriptive t-based 95% confidence intervals (CI). The three repeated observations per patient are presented descriptively as individual trajectories rather than analyzed as independent observations. A multivariable mixed-effects model was not fitted because of the small operative subgroup and the limited availability of potential confounders. Baseline continuous variables were compared using two-sided Welch t-tests, baseline binary variables and readmission/mortality using two-sided Fisher exact tests, and three-category baseline variables (walking aid and treatment period) using two-sided Fisher–Freeman–Halton exact tests. All analyses are exploratory and unadjusted, and no causal treatment effect is inferred. Because the same individuals contributed repeated measurements, cross-sectional Welch tests at each time point are presented solely as descriptive, unadjusted contrasts and must not be interpreted as independent longitudinal treatment effects. No between-group significance test of change was used to infer treatment benefit. Numerical checks of the reported group comparisons for this revision were performed using Python 3.12.14 and SciPy 1.17.0; Fisher–Freeman–Halton *p*-values were verified by enumerating contingency tables with fixed margins.

### 2.6. Ethical Considerations

This retrospective observational study used routinely collected clinical data from existing medical records and involved no study-specific interventions. According to institutional guidance communicated to the investigators, specific ethics committee approval was not required. Individual informed consent was not obtained in accordance with the same institutional guidance. No formal ethics approval number was issued or reported.

## 3. Results

**Figure 1:**
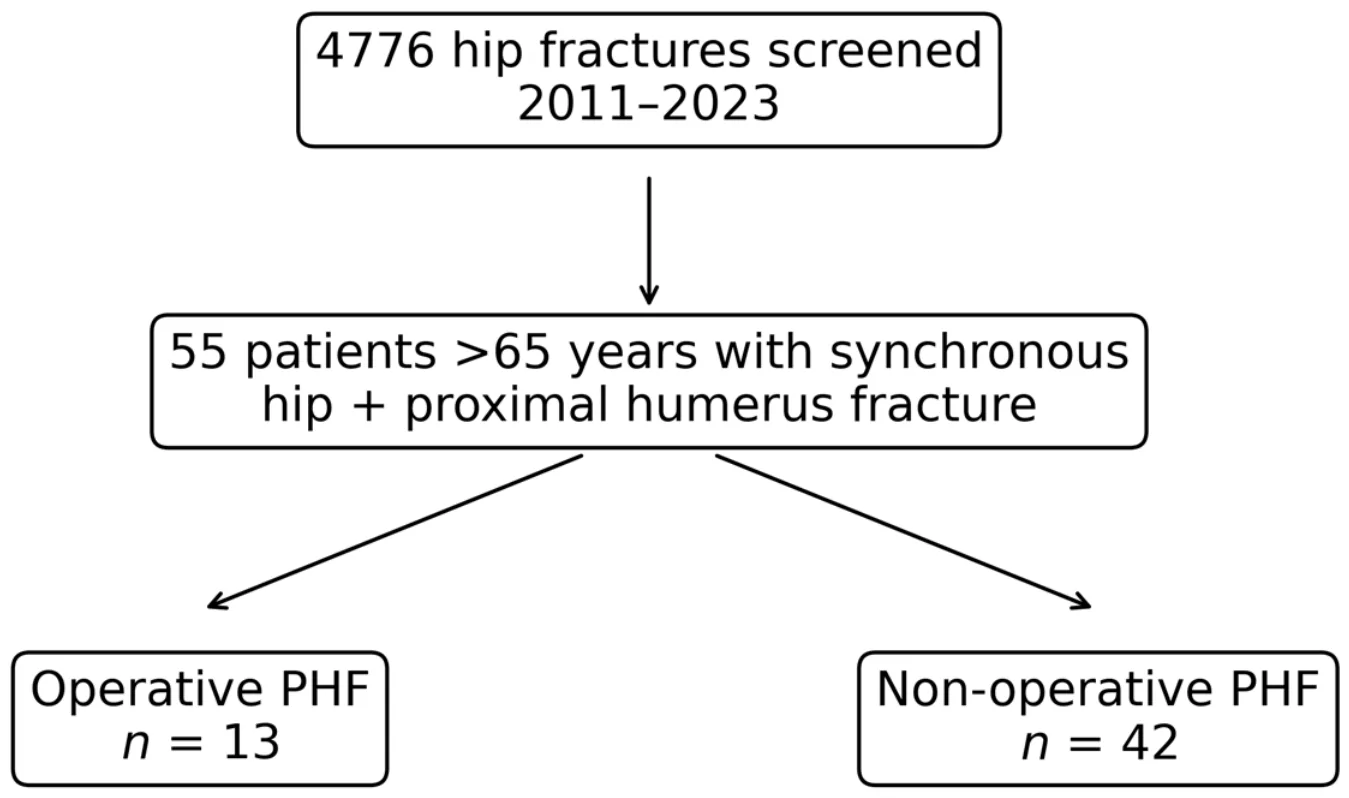
Patient selection flowchart.

**Figure 2:**
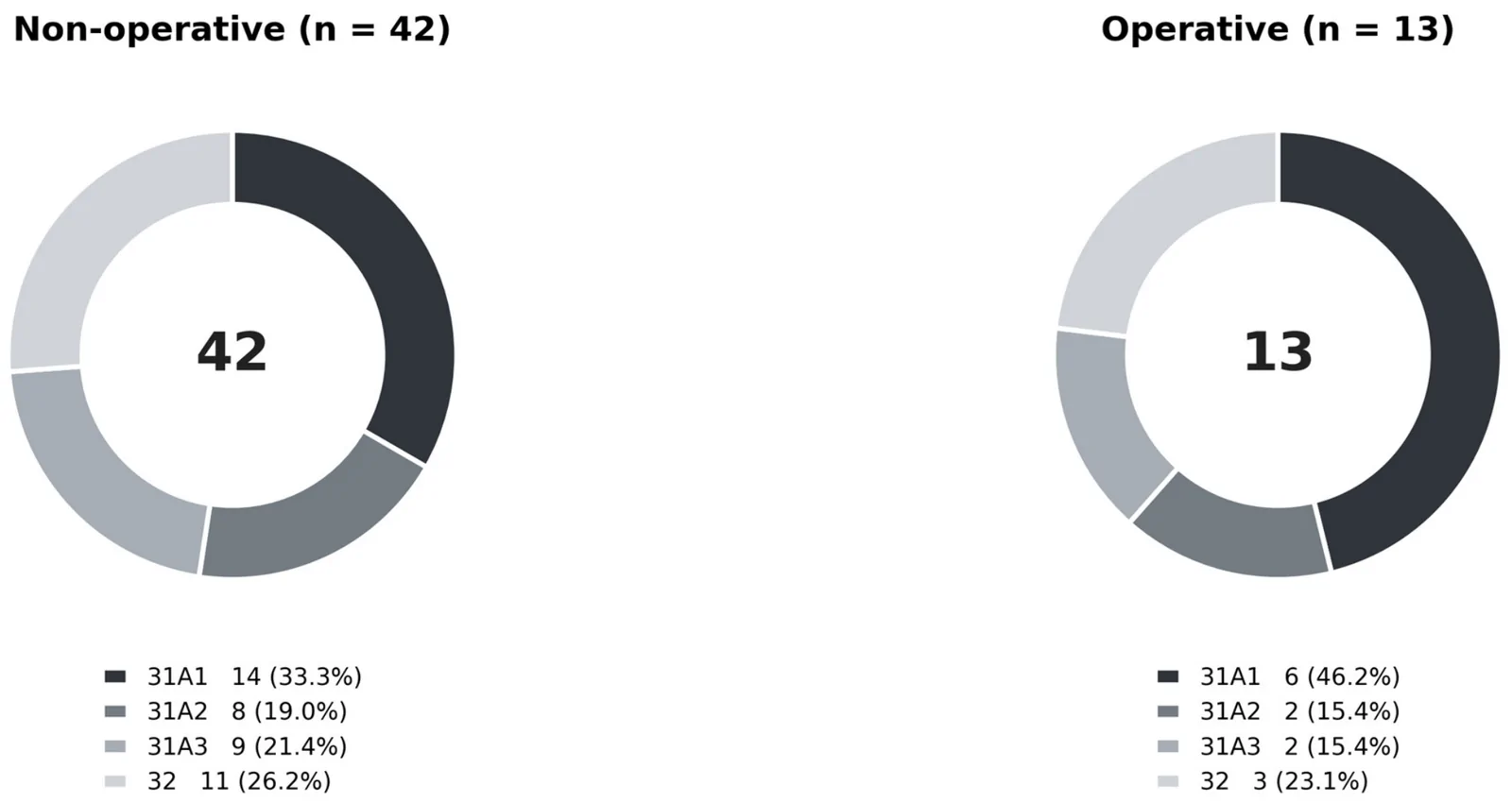
Distribution according to proximal femur fracture type (AO).

**Figure 3:**
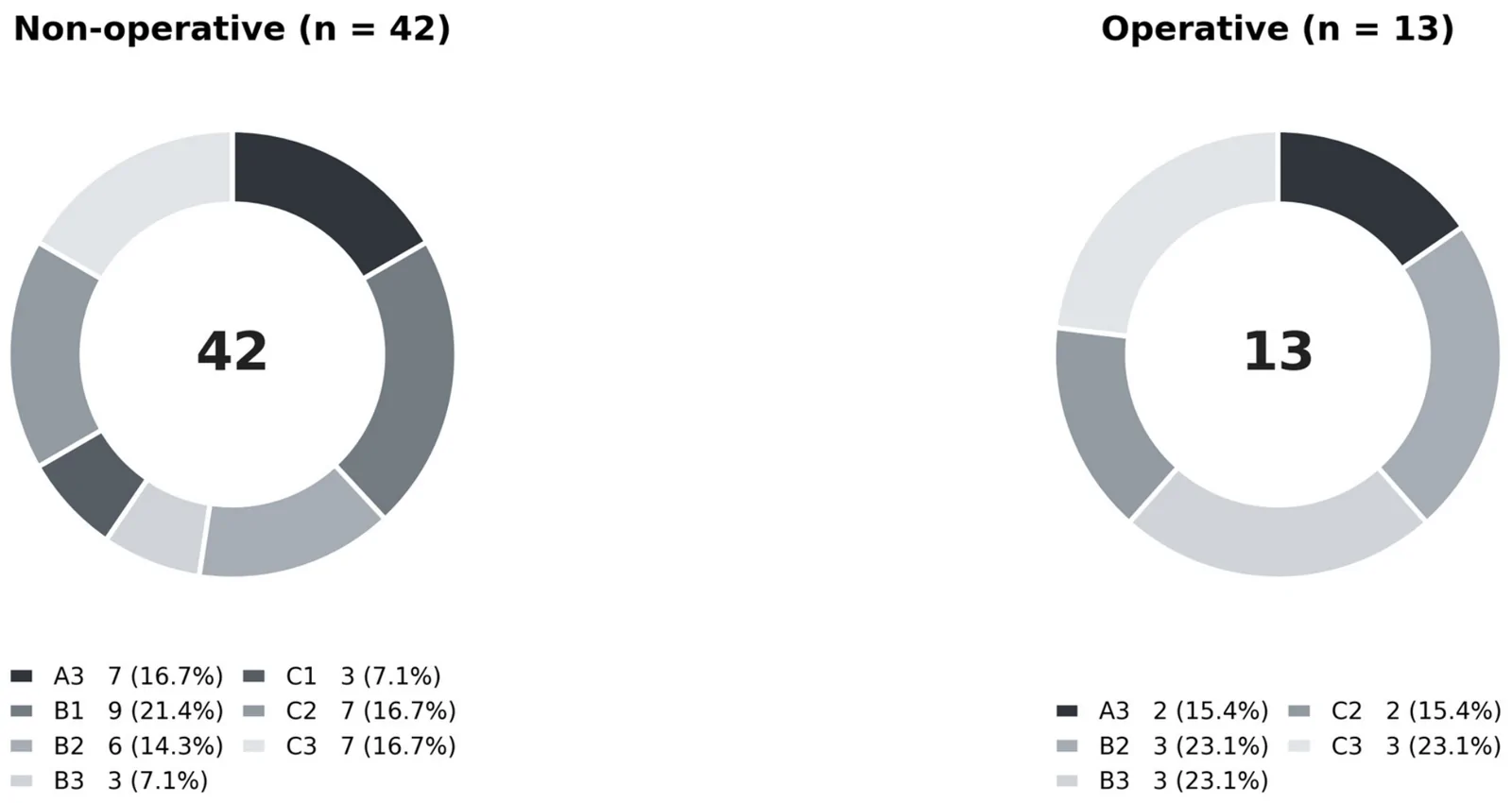
Distribution according to proximal humerus fracture type (AO). AO/OTA proximal humerus categories are displayed by treatment group; counts sum to 13 operative and 42 non-operative patients.

**Figure 4:**
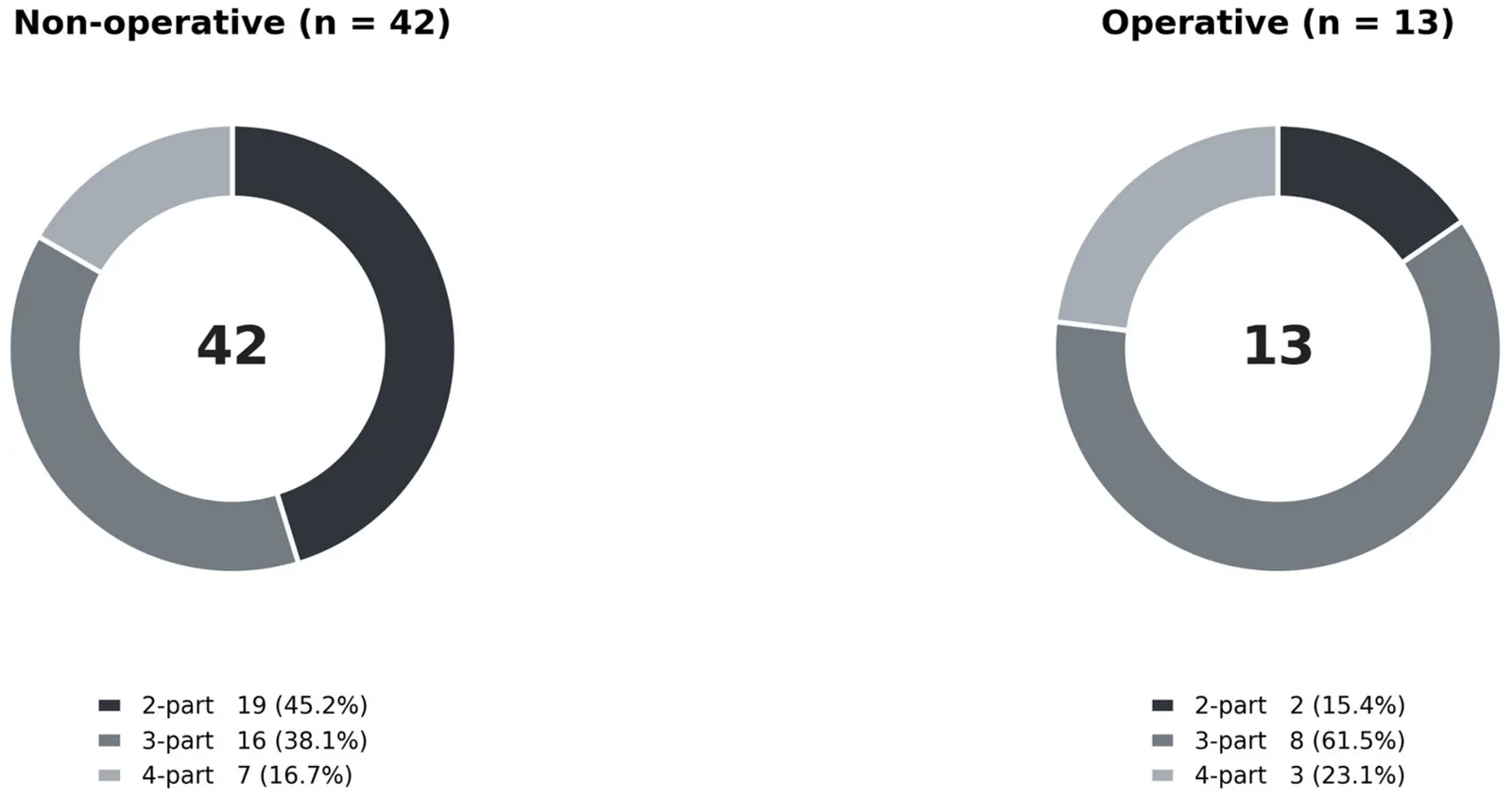
Distribution according to proximal humerus fracture type (Neer). Neer 2-, 3-, and 4-part fracture counts are shown separately for both groups.

**Figure 5:**
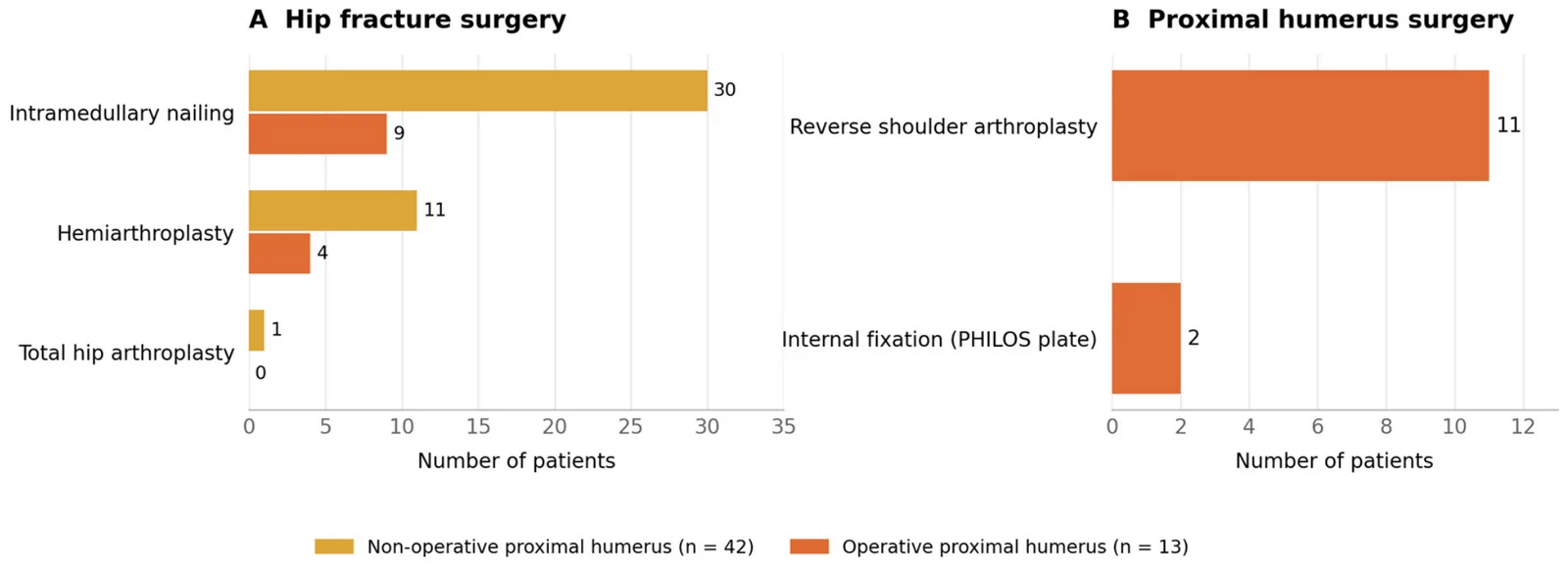
Distribution of operative treatments recorded in the cohort. Eleven operative patients underwent reverse shoulder arthroplasty and two underwent osteosynthesis.

**Figure 6:**
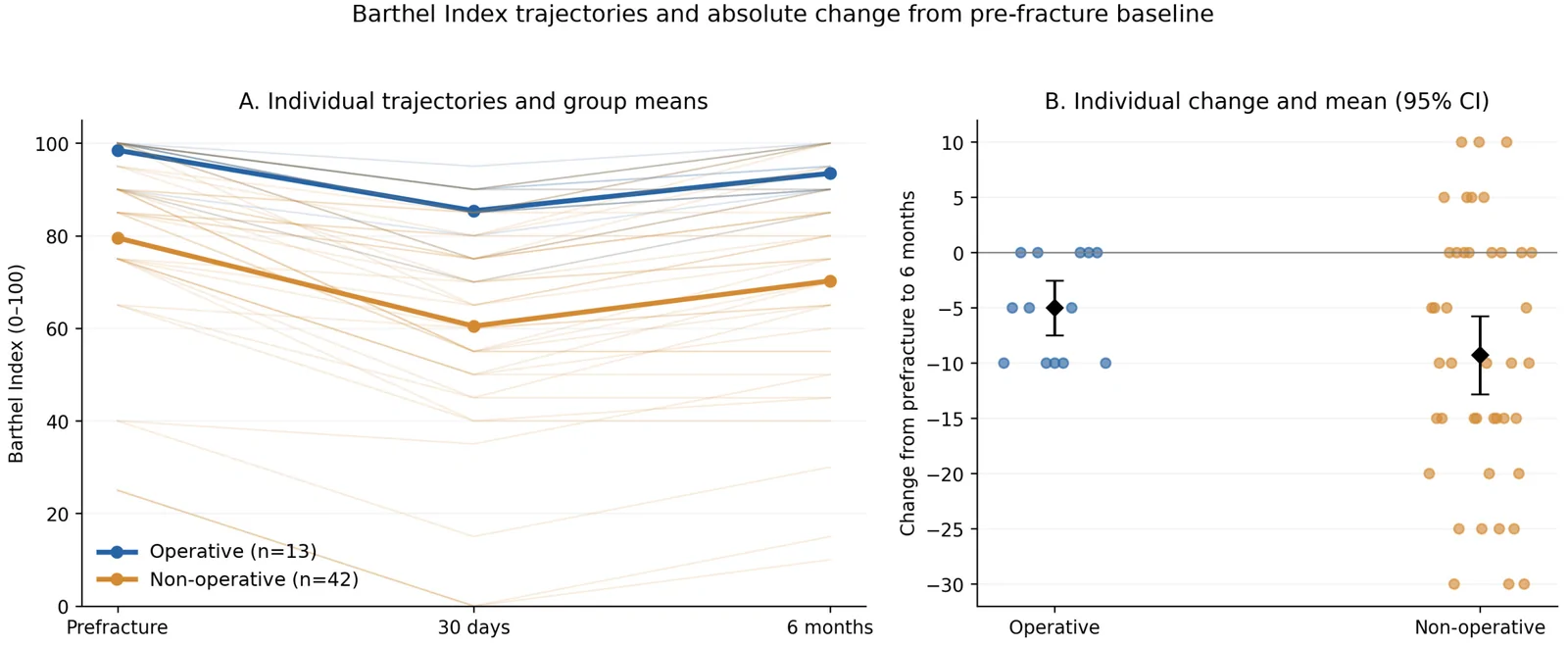
Individual Barthel Index trajectories at pre-fracture baseline, 30 days and six months (**A**), and individual absolute changes from baseline to six months (**B**), stratified by operative versus non-operative proximal humerus management (*n* = 13 and *n* = 42, respectively).

Among 4776 screened hip-fracture presentations, 55 patients had concomitant proximal humerus fractures: 13 underwent operative humerus treatment and 42 were managed non-operatively. Patient selection is summarized in Figure 1. Of the operative cases, 11 underwent reverse shoulder arthroplasty and two underwent osteosynthesis. Pre-fracture Barthel Index was 98.5 ± 3.8 in the operative group and 79.5 ± 20.8 in the non-operative group, indicating substantial baseline imbalance. Table 1 summarizes baseline characteristics and Figure 2, Figure 3 and Figure 4 show fracture classification distributions. The distribution of surgical procedures is shown in Figure 5. Barthel scores at 30 days and six months, and absolute change from baseline, are reported in Table 2 and Figure 6.

Right-hand dominance was present in 12/13 patients (92.3%) in the operative group and 36/42 (85.7%) in the non-operative group (*p* = 1.000). The proximal humerus fracture was ipsilateral to the hip fracture in 11/13 (84.6%) and 34/42 (81.0%) patients, respectively (*p* = 1.000). Among the 13 patients undergoing humeral surgery, 11 (84.6%) underwent both procedures during the same operative session, with hip-fracture surgery performed first and proximal humerus surgery immediately thereafter. In the remaining two patients, humeral surgery was performed 2 and 3 days after hip surgery, respectively. No severe infection or thromboembolic event was recorded among the surgically treated humerus patients. Given the small sample, these observations are descriptive and should not be interpreted as evidence of procedural safety.

Length of hospital stay, readmission, and 12-month mortality are summarized in Table 3. Mean hospital stay was 15 ± 5.33 days in the operative group and 18 ± 9.30 days in the non-operative group (mean difference −3.0 days; Welch t-test calculated from group summaries, *p* = 0.154). Readmission occurred in 1/13 (7.7%) and 3/42 (7.1%) patients, respectively (two-sided Fisher exact *p* = 1.000). Death within 12 months occurred in 0/13 and 2/42 (4.8%) patients, respectively (two-sided Fisher exact *p* = 1.000); both deaths occurred after six months. These analyses are descriptive and cannot establish equivalence or safety.

## 4. Discussion

The principal finding is that patients selected for proximal humerus surgery entered the episode with substantially greater functional independence and remained more independent during follow-up. This baseline difference is not incidental; it reflects the real-world selection process and is also the principal source of confounding. The present data therefore do not demonstrate that humeral surgery itself caused the higher postoperative Barthel scores.

Absolute change from pre-fracture Barthel Index at six months was −5.0 points in the operative group (95% CI −7.8 to −2.2) and −9.3 points in the non-operative group (95% CI −12.9 to −5.7). These are unadjusted, descriptive within-group changes, not estimates of the treatment effect. The bounded Barthel scale and baseline ceiling effect preclude treating the ratio of follow-up to pre-fracture scores as a validated measure of recovery.

The rehabilitation rationale remains clinically relevant. Contemporary hip-fracture rehabilitation guidelines emphasize early mobilization, weight bearing, progressive strengthening, balance, and functional mobility [4,5]. Concomitant upper-extremity injury may interfere with transfers and walking-aid use; systematic-review and recent large-database evidence associate combined injuries with more difficult hospital and rehabilitation courses [6,7,8,9]. However, the 2025 J Clin Med cohort also reported longer hospitalization and more revision surgery among patients whose concomitant upper-extremity fracture was operated on, underscoring the importance of selection rather than routine surgery [8].

Direct evidence for simultaneous hip and proximal humerus fractures is limited. Ganta et al. observed that operative patients were younger and more often community ambulators before injury and reported only a non-significant trend toward return to baseline ambulation [10]. This pattern is closely aligned with the selection issue in our cohort: better baseline function may identify patients in whom restoration of a usable upper limb is considered clinically worthwhile, while simultaneously making unadjusted postoperative comparisons vulnerable to confounding.

Evidence from isolated proximal humerus fractures should also temper causal interpretation. NITEP and PROFHER found no general functional superiority of surgery over non-operative care for many displaced fractures [14,15,16]. A 2025 randomized trial similarly found no advantage of locking-plate fixation at 24 months [18]. Conversely, the 2024 randomized trial by Miquel et al. found better 1-year Constant scores with reverse shoulder arthroplasty in selected patients older than 70 years with complex fractures [17], and longer-term randomized follow-up also supports a potential role for RSA in selected 3- and 4-part fractures [21]. Recent systematic reviews and meta-analyses remain nuanced: selected functional outcomes may favor surgery or RSA, whereas complications and reoperations remain important trade-offs [19,20,21,22,23].

### 4.1. Surgical Strategies and Reverse Shoulder Arthroplasty

In our cohort, 11 of the 13 shoulder operations were reverse shoulder arthroplasties, whereas only two were osteosyntheses. The operative trajectory therefore largely describes patients selected for reverse arthroplasty, but the sample cannot support a procedure-specific analysis. Pooling both procedures should not imply that their indications, restrictions, or expected shoulder recovery are interchangeable. Nor can the outcomes of these 11 patients establish an advantage of reverse arthroplasty over fixation, hemiarthroplasty, or non-operative treatment. Evidence from dedicated shoulder studies is needed to contextualize the treatment mix observed here.

The organization of the two operations is another practical consideration. Eleven patients in our operative group underwent hip and shoulder surgery during the same session, while two underwent shoulder surgery two or three days later. A single session may avoid a second anesthetic episode, whereas a staged approach permits reassessment after hip surgery; these are potential considerations rather than benefits demonstrated by our data. The cohort does not contain sufficient information on operative duration, physiological stress, or reasons for staging to compare these approaches. The absence of recorded severe infection or thromboembolism in 13 patients also cannot establish that a combined session is safer. Decisions about the sequence and timing of shoulder surgery should therefore be individualized, with the hip and shoulder teams considering whether the expected functional gain justifies the additional intervention and whether a workable rehabilitation plan is available. Our results do not identify an optimal sequence or interval.

The distinction between surgical strategies is supported by randomized evidence. NITEP compared non-operative care, locking-plate fixation, and hemiarthroplasty in older adults with displaced three- or four-part fractures and did not demonstrate a functional benefit of either operation at two years [14]. Reverse arthroplasty was not one of its treatment arms, so its findings cannot be treated as a direct test of that procedure. In the DelPhi trial, 124 patients aged 65–85 years with displaced OTA/AO B2 or C2 fractures were randomized to reverse arthroplasty or plate fixation. Mean Constant scores at two years were 68.0 and 54.6, respectively, a difference of 13.4 points (95% CI 6.2–20.6) [28]. This supports reverse arthroplasty over fixation in that selected fracture population, without answering whether either operation improves recovery after concomitant hip fracture.

Hemiarthroplasty provides a further relevant comparison. Sebastiá-Forcada et al. randomized 62 patients older than 70 years with complex proximal humeral fractures to reverse arthroplasty or hemiarthroplasty. Reverse arthroplasty produced better Constant scores and forward elevation; six hemiarthroplasties required conversion to reverse arthroplasty [29]. These results help explain the preference for reverse arthroplasty when replacement is chosen for selected complex geriatric fractures. They do not establish that replacement is preferable for every displaced fracture, or that improved shoulder elevation necessarily permits earlier use of a walking aid. The comparator and the functional task of interest remain essential to interpretation.

Direct comparison with non-operative care provides a more cautious estimate of benefit. In the randomized trial by Miquel et al., 81 patients older than 70 years with three- or four-part fractures were enrolled and 66 completed one-year assessment. Mean Constant scores were 61.24 after reverse arthroplasty and 52.44 after non-operative treatment; the reported difference was 8.84 points (95% CI 1.57–16.11) [17]. This difference was close to commonly considered clinically important thresholds, and two surgical patients experienced major complications, comprising infection and axillary nerve palsy. Thus, a potential shoulder-function benefit must be weighed against operative harm. Neither the endpoint nor the population establishes improved early ambulation in patients also recovering from hip-fracture surgery.

Long-term follow-up by Lopiz et al. also warrants careful interpretation. Among 29 patients available from an original randomized cohort of 62 patients aged at least 80 years, Constant scores favored reverse arthroplasty over non-operative treatment at approximately seven years (62 versus 51 points), whereas pain and SF-12 outcomes did not differ significantly [21]. The small surviving sample and the long interval to assessment limit extrapolation to the immediate rehabilitation needs of a frail hip-fracture patient. Together, these trials suggest that selected patients may gain shoulder-specific function, while leaving uncertainty about the size, timing, and broader functional consequences of that gain.

Reverse arthroplasty may be attractive when a complex fracture is difficult to reconstruct and recovery through fixation is uncertain. Its rationale is to obtain usable shoulder function despite compromised proximal humeral anatomy. However, it does not remove the importance of the tuberosities. In a multicenter cohort of 420 patients, Ohl et al. associated anatomic greater-tuberosity healing with better elevation, external rotation, and subjective outcomes; tuberosity excision was associated with more instability [32]. Boileau et al. similarly reported better elevation and external rotation after tuberosity healing [33]. These observational findings support attention to reconstruction and healing even with a reverse implant. For patients needing their arms during transfers, eventual functional capacity and the protection required during recovery must both be considered.

### 4.2. Rehabilitation and Treatment Selection

Non-operative treatment remains a central strategy in geriatric PHF care [24]. Rehabilitation timing and organization are also relevant [25,26], and frailty may help predict complications and functional recovery [27]. Thus, fracture morphology alone is insufficient; baseline autonomy, cognition, frailty, comorbidity, rehabilitation demands, and patient goals should be considered together.

A potential longer-term shoulder benefit must be distinguished from permission for immediate weight bearing. In a series of reverse arthroplasties for patients with weight-bearing shoulders, Levy et al. restricted pushing up from a wheelchair for six weeks [34]. Most shoulders in that series were treated for conditions other than acute fracture, so the findings provide indirect guidance rather than a protocol for synchronous hip and humerus fractures. Nevertheless, they demonstrate that an implanted reverse prosthesis should not automatically be regarded as ready for unrestricted loading. The early rehabilitation plan must specify permitted use rather than infer it from the name of the procedure.

For a patient with a concomitant hip fracture, the practical question is therefore whether the proposed shoulder strategy is compatible with the assistance and mobility aids required during recovery. As a clinical implication, the team should distinguish shoulder motion, light functional use, and loading during a transfer or gait. These tasks impose different demands and may become feasible at different times. A patient who needs both arms to rise from a chair may require substantial assistance while the shoulder is protected, even if pain and passive motion improve. Device selection and transfer techniques should consequently be agreed with the rehabilitation team and reassessed as healing progresses. Our study did not measure these task-specific milestones, and the proposed implications should not be read as observed mechanisms of the Barthel changes.

Preserved pre-fracture independence may increase the practical value a patient places on recovering a useful shoulder, but it cannot serve as a stand-alone indication for reverse arthroplasty. Fracture reconstructibility, expected shoulder recovery, ability to participate in rehabilitation, and the patient’s priorities need to be considered alongside the additional operative burden. The direct combined-injury cohort reported by Ganta et al. reinforces this caution: surgical patients were younger and more often community ambulators, while the higher proportion returning to baseline ambulation at six months did not reach statistical significance [10]. Our findings show a similar selection problem. They support making baseline function explicit in the discussion, without establishing which patient would recover better if assigned to another treatment.

The timing of recovery also matters when interpreting the available shoulder evidence. Outcomes measured at one or two years may be relevant to sustained independence but cannot establish what a patient can do during the first postoperative weeks. In our cohort, the 30-day and six-month Barthel assessments capture global recovery at two clinically useful intervals, but they do not identify when assisted transfers, independent walking, or unrestricted upper-limb loading became possible. Future studies of reverse arthroplasty in this setting should record these milestones directly, together with pain during walking-aid use, assistance required for personal care, and discharge destination. This would make the rehabilitation rationale testable rather than infer it from a later shoulder score.

Non-operative care should likewise be understood as an active rehabilitation pathway. Østergaard et al. found no clinically relevant advantage of weekly supervised exercises over home exercises in a randomized trial of 72 older adults with isolated, displaced two-part fractures [26]. That result should not be assumed to apply to patients simultaneously rehabilitating a hip fracture, who may need help with mobility and daily activities beyond the shoulder exercise programme. The appropriate intensity and setting of support depend on the patient’s combined impairments and available assistance. Because individual rehabilitation exposure was not quantified in our cohort, we cannot determine whether differences in therapy, protection, or support contributed to the observed functional trajectories.

Although a Barthel Index above 90 conventionally describes preserved or near-independent basic activities of daily living [30,31], it is not a validated indication for proximal humerus surgery. The operative group in this cohort had a mean pre-fracture Barthel Index of 98.5 and a six-month mean of 93.5. The findings suggest that baseline independence influenced treatment selection; they do not establish a numerical treatment threshold or causal benefit. No receiver operating characteristic (ROC) analysis or external validation was performed.

The Barthel Index measures global activities of daily living and is strongly influenced by hip function, cognition, frailty, and the overall rehabilitation pathway [1,2,3,27,35]. Because shoulder-specific measures such as Constant, the American Shoulder and Elbow Surgeons (ASES) score, the Quick Disabilities of the Arm, Shoulder and Hand (QuickDASH) questionnaire, pain, and range of motion were not consistently available, the observed trajectory cannot be attributed specifically to restoration of shoulder function.

Finally, the small number of readmissions and deaths means that the absence of statistically detectable differences cannot establish equivalence or safety. Larger multicenter cohorts with standardized shoulder outcomes and prospective capture of baseline function are required. Future investigations should prospectively record fracture morphology, comorbidity and frailty, treatment-selection rationale, upper-limb weight-bearing restrictions, rehabilitation adherence, and validated shoulder-specific outcomes alongside Barthel assessments. A multicenter design with prespecified confounder adjustment and sufficient overlap between treatment groups would be needed before any comparative effectiveness claim could be considered. In clinical practice, these data may help frame multidisciplinary discussions of rehabilitation needs, but should not be used to determine surgery from a Barthel threshold alone.

### 4.3. Limitations

This study has several limitations. First, its retrospective, non-randomized design introduces substantial treatment-selection bias and residual confounding because pre-fracture function contributed to the decision to operate. Second, the sample is small, particularly the operative subgroup (*n* = 13), limiting statistical power and preventing reliable multivariable adjustment or a stable mixed-effects model incorporating clinically relevant covariates. Third, potentially influential baseline factors, including American Society of Anesthesiologists (ASA) physical status classification, formal frailty scores, comorbidity burden and nutritional status, were not systematically available by treatment group and therefore could not be included in the comparative analysis. Hip-fracture procedure and detailed humeral fracture-severity distributions were also not available as sufficiently complete patient-level, treatment-stratified covariates for adjustment; descriptive fracture classification figures do not substitute for comparable baseline covariate measurement. Although age, residence, walking-aid use and treatment period are reported, these variables do not eliminate confounding. Fourth, pre-fracture functional status was obtained from clinical records based on routine geriatric assessment, and individual rehabilitation adherence could not be quantified beyond the usual care pathway described above. Fifth, shoulder-specific outcomes (e.g., Constant-Murley, ASES, QuickDASH, pain and range of motion) were not consistently available; therefore, Barthel trajectories reflect global autonomy rather than shoulder-specific recovery. Finally, the low number of readmissions and deaths precludes conclusions regarding equivalence or safety.

The predominance of reverse arthroplasty within the operative group is an additional limitation of interpretation. With only two osteosyntheses and no hemiarthroplasties, the cohort cannot compare surgical strategies or identify procedure-specific recovery profiles. Implant characteristics, tuberosity healing, and the actual timing of permitted upper-limb loading were not analyzed as separate determinants of outcome. The present findings therefore should not be used to select a particular implant or prescribe a loading schedule.

## 5. Conclusions

Patients selected for operative treatment of concomitant proximal humerus fracture had greater pre-fracture independence and higher Barthel scores during follow-up than those selected for non-operative care. Both groups experienced an absolute decline from pre-fracture independence at six months. The differences must be interpreted in the context of substantial baseline imbalance, clinical treatment selection, and the limited sample. This study does not establish that operative treatment improves functional recovery. The observed trajectories are useful for characterizing the rehabilitation needs of clinically selected patient groups, but should not be used to choose operative management on the basis of a Barthel threshold.

## Figures and Tables

**Table 1:** Baseline characteristics.

Variable	Operative PHF	Non-Operative PHF	*p*-Value
Patients	13	42	Not applicable
Female sex	9/13 (69.2%)	35/42 (83.3%)	0.427
Independent ambulation	12/13 (92.3%)	23/42 (54.8%)	0.019
Cognitive impairment	3/13 (23.1%)	14/42 (33.3%)	0.733
Right-hand dominance	12/13 (92.3%)	36/42 (85.7%)	1.000
Ipsilateral proximal humerus fracture	11/13 (84.6%)	34/42 (81.0%)	1.000
Pre-fracture Barthel Index	98.5 ± 3.8	79.5 ± 20.8	<0.001
Age, years	78.0 ± 6.1	81.9 ± 7.3	0.067
Residence			0.255
Community dwelling	12/13 (92.3%)	31/42 (73.8%)	
Institutional residence	1/13 (7.7%)	11/42 (26.2%)	
Walking aid			0.092
None	10/13 (76.9%)	18/42 (42.9%)	
Cane	2/13 (15.4%)	11/42 (26.2%)	
Walker	1/13 (7.7%)	13/42 (31.0%)	
Treatment period			0.678
2011–2015	3/13 (23.1%)	14/42 (33.3%)	
2016–2020	5/13 (38.5%)	16/42 (38.1%)	
2021–2023	5/13 (38.5%)	12/42 (28.6%)	

Table 1 note: Values are *n* (%) or mean ± SD. Between-group comparisons used two-sided Welch t-tests for continuous variables, Fisher exact tests for binary variables, and Fisher–Freeman–Halton exact tests for three-category variables (walking aid and treatment period). For residence, walking aid and treatment period, the *p*-value in the group heading represents the overall comparison across all categories; blank category cells do not indicate missing analyses. The patient counts are descriptive and were not compared statistically. All tests were two-sided. PHF, proximal humerus fracture; SD, standard deviation.

**Table 2:** Barthel Index trajectory and descriptive change from baseline.

Time Point	Operative PHF	Non-Operative PHF	*p*-Value	Δ Operative	Δ Non-Operative
Pre-fracture	98.5 ± 3.8	79.5 ± 20.8	<0.001	Reference	Reference
30 days	85.4 ± 6.9	60.5 ± 23.9	<0.001	−13.1 (95% CI −16.2 to −9.9)	−19.0 (95% CI −22.2 to −15.9)
6 months	93.5 ± 5.2	70.2 ± 25.0	<0.001	−5.0 (95% CI −7.8 to −2.2)	−9.3 (95% CI −12.9 to −5.7)

**Table 3:** Length of stay, readmission, and mortality.

Outcome	Operative PHF	Non-Operative PHF	*p*-Value
Hospital stay, days	15 ± 5.33	18 ± 9.30	0.154
Readmission	1/13 (7.7%)	3/42 (7.1%)	1.000
Deaths	0/13	2/42 (4.8%)	1.000

## Data Availability

The data supporting the findings of this study are not publicly available due to patient confidentiality and institutional data protection requirements. Requests for access to anonymized data may be considered by the corresponding author, subject to institutional authorization and applicable data protection regulations.
